# Supplementary material for: Unraveling the genomic complexity of secretion systems in the most virulent Xanthomonas arboricola pathovars
Source: PLoS One. 2025 Sep 19;20(9):e0332834. doi: 10.1371/journal.pone.0332834 (PMC12448352; doi:10.1371/journal.pone.0332834)

**S1 Fig.** Additional type V secretion systems found with TXSScan. **A.** Homologs to UQQ05999.1 from IVIA 2626.1. **B.** Homologs to UQQ08703.1 from IVIA 2626.1. **C.** Homologs to UQQ06620.1 from IVIA 2626.1.

**A**


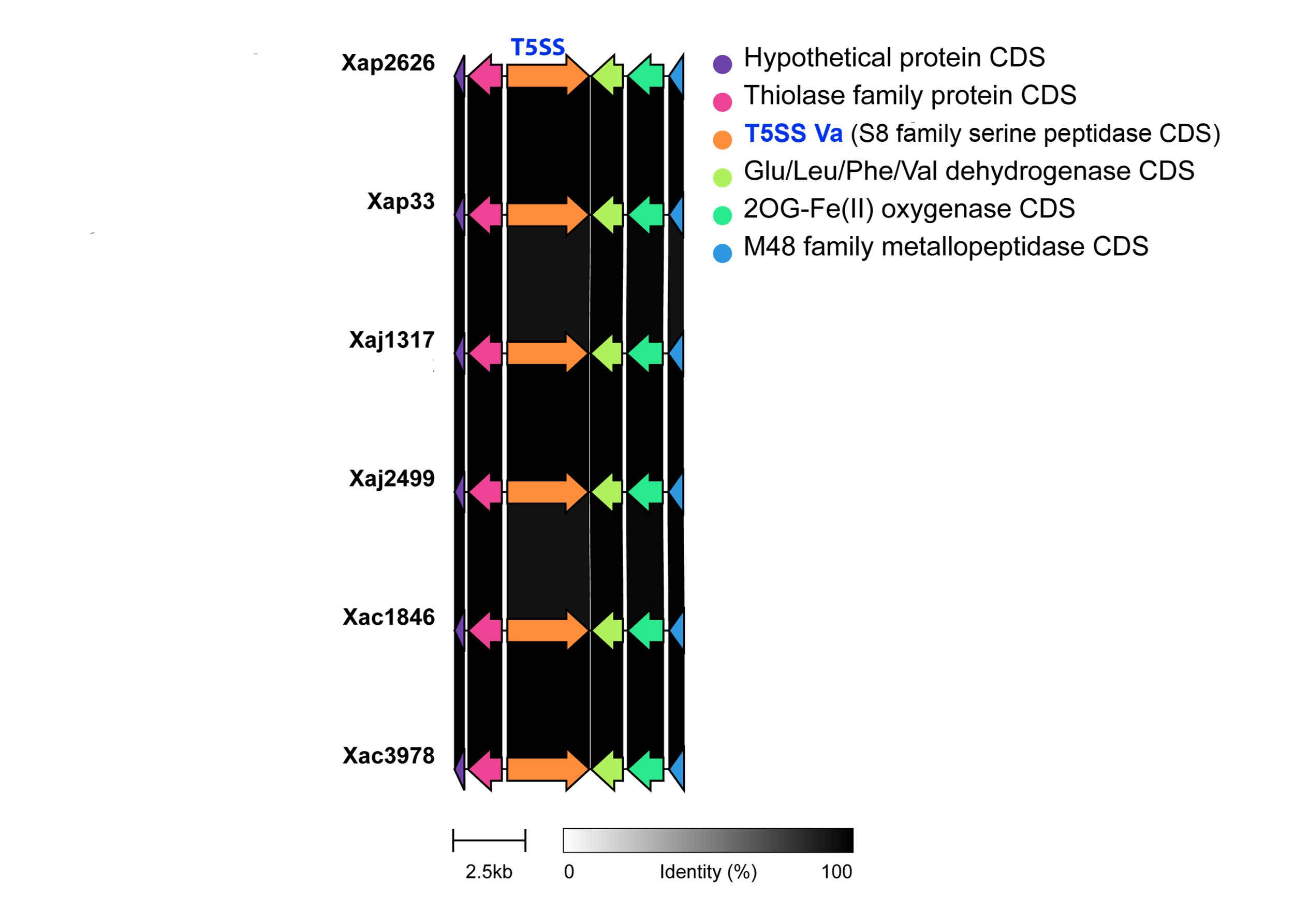


**B**


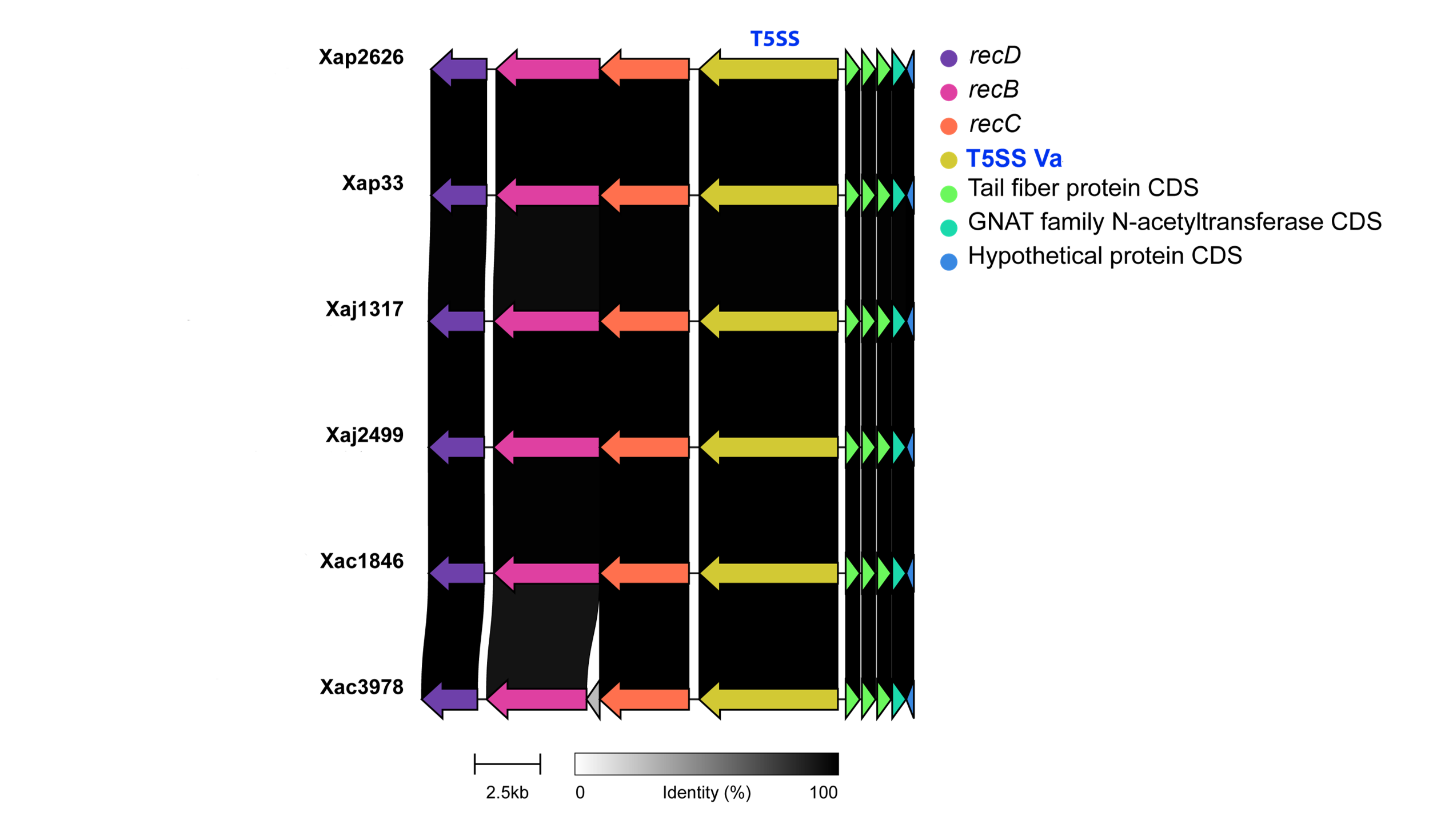


**C**


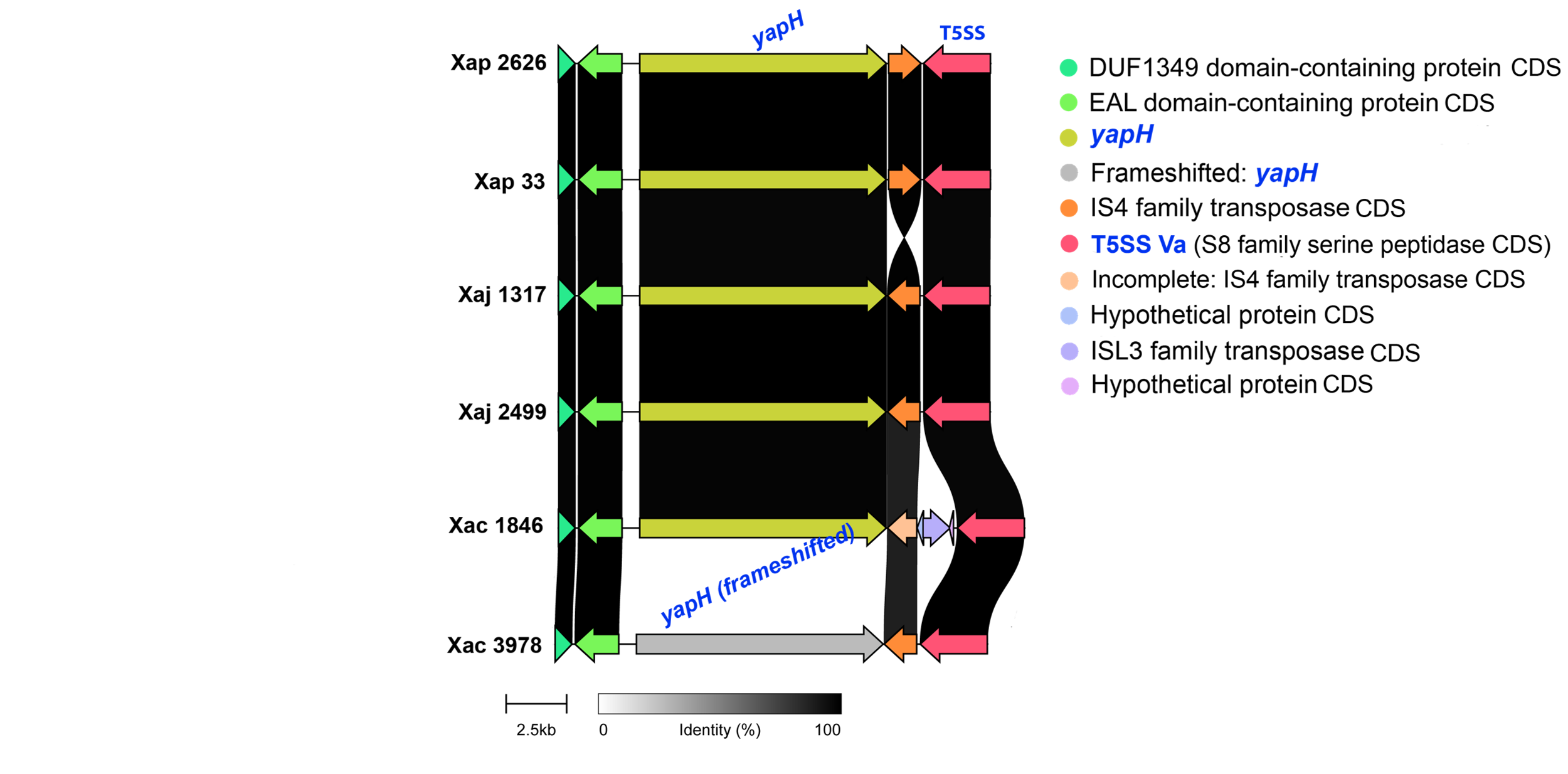

Supplement: S1 Fig — A. Homologs to UQQ05999.1 from IVIA 2626.1. B. Homologs to UQQ08703.1 from IVIA 2626.1. C. Homologs to UQQ06620.1 from IVIA 2626.1. (DOCX) [file pone.0332834.s003.docx]
