# Supplementary material for: Unraveling the genomic complexity of secretion systems in the most virulent Xanthomonas arboricola pathovars
Source: PLoS One. 2025 Sep 19;20(9):e0332834. doi: 10.1371/journal.pone.0332834 (PMC12448352; doi:10.1371/journal.pone.0332834)
Supplement: S2 Fig — PctB from Xanthomonas arboricola strains present the conserved Cys, His and Asp residues (yellow) that are predicted to form the active site and the conserved Gln residue (green) that may form the oxyanion hole. Xap 2626 = IVIA 2626.1, Xap 33 = CITA 33, Xac 3978 = IVIA 3978. Based on Luu et al., 2019. (DOCX) [file pone.0332834.s004.docx]

**S2 Fig.** Alignment of cysteine (C) and histidine (H) motifs of the C39 peptidase domain of PctB sequences from IVIA 2626.1, CITA 33, IVIA 3978 and other peptidase-containing ATP-binding cassette transporters described in literature (Luu et al., 2019). PctB from *Xanthomonas arboricola* strains present the conserved Cys, His and Asp residues (yellow) that are predicted to form the active site and the conserved Gln residue (green) that may form the oxyanion hole. Xap 2626=IVIA 2626.1, Xap 33=CITA 33, Xac 3978=IVIA 3978. Based on Luu et al., 2019.

**
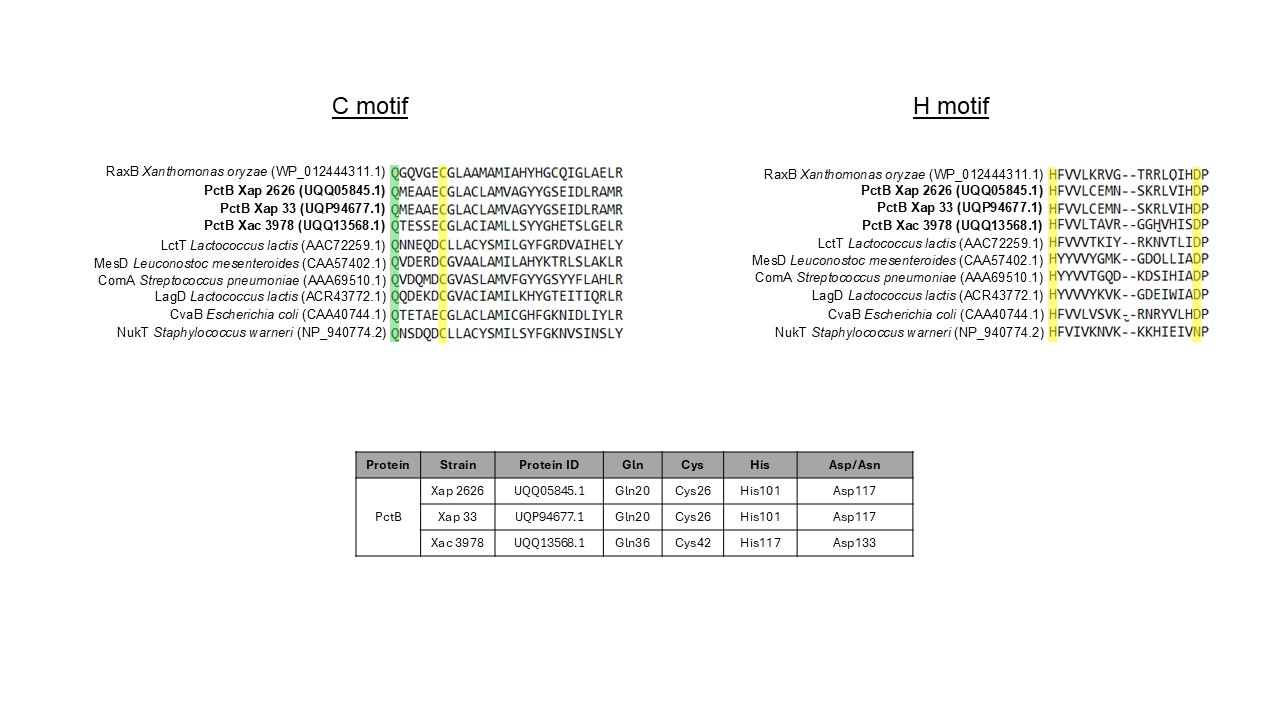
**
