# Supplementary material for: Unraveling the genomic complexity of secretion systems in the most virulent Xanthomonas arboricola pathovars
Source: PLoS One. 2025 Sep 19;20(9):e0332834. doi: 10.1371/journal.pone.0332834 (PMC12448352; doi:10.1371/journal.pone.0332834)
Supplement: Attachment — Submitted filename: Response to Reviewers.docx [file pone.0332834.s006.docx]

**Rebuttal Letter**

We are highly grateful to the reviewers for their thorough and valuable suggestions for improving the manuscript, all of them adressed

**Editor:**

***“Your manuscript presents an engaging comparative genomics study focused on Xanthomonas and the diversity of its secretion systems. Following peer review, both reviewers concurred that the manuscript would benefit from further refinement, particularly regarding the definition of homologous genes and the application of more robust sequence alignment tools beyond BLAST to enhance validation. I do agree with them.”***

We appreciate the positive comments regarding the article and the work carried out. Concerning the definition of homologous genes, as suggested by one of the reviewers, we have added information in the text about the parameters used to determine homology and emphasized that the identification was based not only on sequence similarity criteria.

***Journal Requirements:***

***Please ensure that your manuscript meets PLOS ONE's style requirements, including those for file naming. The PLOS ONE style templates can be found at***

OK

***Thank you for stating the following financial disclosure: [This study was supported by PID2021-123600OR-C41 funded MICIU / AEI /10.13039/501100011033/, by “ERDF A way of making Europe” and PRE2019-090846 “ESF Investing in your future”.].***

***Please state what role the funders took in the study.  If the funders had no role, please state: ""The funders had no role in study design, data collection and analysis, decision to publish, or preparation of the manuscript."***

We have included this amended Role of Funder statement in the text and in our cover letter.

***We note that there is identifying data in the Supporting Information file < Supplementary_Table_1>. Due to the inclusion of these potentially identifying data, we have removed this file from your file inventory. Prior to sharing human research participant data, authors should consult with an ethics committee to ensure data are shared in accordance with participant consent and all applicable local laws.***

We do not understand what is being requested here. The table in question contains data related to bacterial strains and the bibliographic references that support it. There is no data subject to protection, and of course, nothing related to humans.

**Reviewers' comments:**

***Reviewer #1: This is a well-conceived and methodologically solid comparative genomics study that explores the diversity of secretion systems (T1SS–T5SS) across six closed genomes representing the most virulent Xanthomonas arboricola pathovars: pruni, juglandis, and corylina. The work contributes meaningfully to the field of plant pathology by offering insights into the genomic architecture underlying host specificity and virulence potential.***

Thanks for your positive comments.

***The Methods section states that protein hits were considered homologs when they satisfied three criteria: E-value < 0.001, query coverage >80%, and sequence identity >40%. The authors cite Pearson (2013) [DOI: 10.1002/0471250953.bi0301s42] in support of this threshold, which is appropriate given that the referenced work emphasizes the importance of statistical significance (e.g., E-values and bit scores) over raw percent identity when inferring homology.***

***However, I recommend that the authors explicitly elaborate on this rationale in the manuscript, especially given the potential concern that a 40% identity threshold may allow the inclusion of distant homologs with uncertain functional conservation, even when high query coverage is used. This is particularly important for secretion system effectors, where minor sequence divergence may translate into major functional differences.*
*To improve clarity and strengthen the reliability of the results, I suggest the authors:
Briefly summarize Pearson’s argument (e.g., that statistical measures are more reliable than identity percentages alone in homology inference).***

***Clarify whether domain conservation (e.g., InterProScan) or other functional annotations were used to validate low-identity hits.***

Thank you for the comment that helps clarify the focus of our analysis. We have modified the text according to your suggestion and included the argument used by the authors of the cited manuscript. Indeed, we have encompassed in the text the parameters employed to determine homology and have clarified that the identification relied on more than just sequence similarity criteria.

***Minor Changes***

***-Typographical edits:***

***Line 52: "Thes pathovars" → "These pathovars"***

Ok, corrected.

***Lines 69–70: Consider rephrasing slightly for clarity: “Secretion systems are complex molecular machineries…”***

Ok, we’ve rephrased it. However, because Type IV and VI secretion systems target the cytoplasm of other bacteria rather than the host or extracellular space, we decided to retain that part of the sentence to reflect this.

***Line 156: “appeared to have been interrupted…” to “were likely disrupted…”***

Ok, changed.

***Optional addition:***

***-A brief sentence in the conclusions and/or discussing how the findings could guide future experimental design (e.g., validation of T1SS function or virulence assays) would help bridge the gap to applied research.***

Thanks for the comment, we have added a sentence regarding. We have added a sentence to this effect at the end of the conclusions, reinforcing the idea that the results may serve as a basis for future functional studies and contribute to the control of phytopathogenic bacteria.

***Reviewer #2: In this manuscript, Cuesta-Morrondo and colleagues present a comparative genomic analysis of six previously sequenced Xanthomonas arboricola genomes. Given the well established role of secretion systems in bacterial pathogenicity, the authors focus their analysis on the occurrence and genomic organization of type I, II, III, and V secretion systems across Xa pathovars of agricultural relevance.The authors provide a systematic description of the presence/absnece patterns and genomic arrangement of the aforementioned secretion systems. While primarily descriptive in nature, with the authors limiting their analysis to the identification of gene clusters encoding these secretion systems, the study offers insights that will be of interest to researchers investigating Xanthomonas pathogenicity.***

***Taking advantage of available high quality genomes, the authors report some interesting findings, including the identification of a TALE effector, which is often overlooked in draft genome analyses due to its repetitive structure. The authors also provide a detailed description of the distribution of distinct type III effectors across the analyzed genomes which may, at least partially, help explain differences in pathogenic profiles and host-pathogen interactions among X. arboricola pathovars.***

We sincerely thank you for your positive feedback and for recognizing the significance and relevance of the work presented.

***Major comment:***

***The key methodological limitation of the manuscript is the approach taken to detect secretion systems, as the authors rely only on BLAST searches, mainly against known components of secretion systems from Xanthomonas genomes. While effective for detecting conserved systems, this method may fail to identify divergent or lineage-specific secretion systems, therefore limiting the depth and completeness of the analysis. To address this limitation, I strongly recommend the use of TXSScan (***[***https://github.com/macsy-models/TXSScan***](https://github.com/macsy-models/TXSScan)***), a dedicated tool for the comprehensive identification of bacterial secretion systems. TXSScan integrates HMM profile searche and synteny criteria, offering enhanced sensitivity compared to BLAST based methods alone. Since the primary claim of the study concerns the diversity and organization of secretion systems, reanalysis using TXSScan would strengthen the conclusions and, potentially, could reveal additional systems not detected with the current approach.
Given that TXSScan is relatively simple and straightforward to implement, this reviewer recommend reanalyzing the genomes using TXSScan to cross-validate, and potentially expand,the identification of secretion systems in the studied Xa genomes. This would ensure the completeness and robustness of the findings prior to publication.***

Thank you for the suggestion. We have reanalyzed our data using the proposed tool and confirmed the results in most cases. The outcomes obtained with this tool have been cited throughout the text for each of the gene groups studied, thereby completing the work and following the reviewer's recommendation. Moreover, we have included the reference for the tool and an additional supplementary material to compare results between BLAST and TXSScan.

***Minor comments:***

***Line 34: "variation was" instead of "variation were."***

Ok, changed

***line 113: Review and correct citation format (Cuesta-Morrondo et al., 2022).***

Ok, changed

***line 189: The authors mention that xcsK is frameshifted…consider claryfing here whether xcsK is essential for T2SS function, especially for readers unfamiliar with the system nomenclature in Xanthomonas.***

Thanks for your comment. While there are some studies suggesting possible functions for the Xcs T2SS, its role remains unclear. Therefore, it is not possible to assess if xcsK is essential. We have included some information in the main text regarding this issue

***line 190: Redaction needs to be double checked...consider rephraseing “Moreover, orthologs of eight Xanthomonas citri pv. citri experimentally validated T2SS substrates [42] were” to “Moreover, orthologs of eight experimentally validated T2SS substrates from Xanthomonas citri pv. citri were”***

Ok, changed. We also revised the whole text.

***Line 396: Rephrase: "Xac strains harbored two XAC2853 orthologs" to "...harbored two homologs of XAC2853", as the presence of two related genes within the same genome indicates paralogs, not orthologs.***

Ok, changed

***Fig. 1A: Labels are too small to read.***

The figure has been modified to increase the size of the labels.

***Fig. 3 and 4: These both relate to T3SS and could be merged into a single figure for clarity and consistency***

By merging 4 with 6b, we preferred to keep 3 and 4 separate for greater clarity for the reader.

***Fig. 5: Again…gene labels are too small, especially in panel 5C***

Figure was modified to increase label size.

***Fig. 6A: Clarify the meaning of the lighter blue boxes in the figure legend. I guess they represent predicted non-functional clusters (due to frameshifts) but this should be explicitly stated at least in the figure legend.***

The legend was modified to clarify that they are incomplete secretion systems, containing only some of the components.

***Fig. 6B: This panel conveys essentially the same information as Figure 4A. Consider replacing 4A with 6B to avoid redundancy***

Part of Figure 6 was merged with Figure 4, but we believe that the summary presented in the new Figure 6 is quite informative for the reader and it should be preserved
