# Supplementary material for: Unraveling the genomic complexity of secretion systems in the most virulent Xanthomonas arboricola pathovars
Source: PLoS One. 2025 Sep 19;20(9):e0332834. doi: 10.1371/journal.pone.0332834 (PMC12448352; doi:10.1371/journal.pone.0332834)
Supplement: Attachment — Submitted filename: Response to Reviewers 2.docx [file pone.0332834.s007.docx]

**Rebuttal Letter**

Once again we thanks to the reviewers for their interest on the manuscript and their final suggestions for improving it. All of them were addressed.

**Editor:**

***“The requested revisions have been satisfactorily addressed, as confirmed by both reviewers. Nonetheless, one reviewer and I concur that the manuscript’s clarity would benefit from refining certain statements—particularly within the figure captions—to make them more self-explanatory and comprehensive. You are encouraged to implement these improvements, after which I will be able to accept the article without requiring further revision.”***

We appreciate again the positive comments regarding the article and the work carried out and we have addressed the final suggestions and corrections from one of the reviewers.

**Reviewers' comments:**

***Reviewer #2:***

***The figure legends are, in general, too brief and would benefit from being more descriptive. The legend of Figure 4 is particularly vague: “B. T3Es Venn diagram.” … a Venn diagram of what? Please elaborate (e.g. “Venn diagram showing the shared and unique repertoires of…”).***

Indeed the reviewer is right. We have modified all the legends according to such comment in this new version

***The sentence “TXSScan also supported the T3SS in all the strains under study”....should read "TXSScan also supported the PRESENCE OF the T3SS in all…" ?***

Thank you for the comment. The sentence was modified according to reviewer´s comment.
